# Resident Perceptions of Simulcast Teaching: A Qualitative Study

**DOI:** 10.1177/23821205241281350

**Published:** 2024-09-19

**Authors:** Rachel S. Casas, Jennifer L. Cooper, Susan A. Glod, Eliana V. Hempel

**Affiliations:** 1 12310 Division of General Internal Medicine, Penn State Milton S. Hershey Medical Center, Hershey, PA, USA; 2 Division of Hospice and Palliative Medicine, 12311 Penn State Milton S. Hershey Medical Center, Hershey, PA, USA

**Keywords:** educational technology, educational models, E learning, graduate medical education, internal medicine

## Abstract

**OBJECTIVES:**

During the COVID-19 pandemic, medical education programs were challenged to optimize learning while balancing social interaction with exposure risk. In response, our internal medicine (IM) residency program transitioned to “simulcast” educational sessions. In simulcast sessions, multiple small groups of learners met in person in separate rooms and connected to the large-group session via videoconferencing. This qualitative study describes IM residents’ perceptions regarding the advantages and disadvantages of learning in simulcast compared to virtual and in-person settings during the pandemic.

**METHODS:**

Categorical IM residents at Penn State during the academic year 2020-2021 were invited to participate. Eligible residents participated in one 30-min virtual, semistructured focus group. We used inductive thematic coding to analyze resident responses.

**RESULTS:**

Forty-eight percent (*n* = 29/60) of invited residents participated in focus groups. In the simulcast setting, participants felt more accountability to participate in their small groups compared to a larger group or virtual setting. Educational experiences varied based upon facilitator skill level. Overall, in-person settings were preferred to virtual, when possible, due to increased social connection. Respondents identified educator enthusiasm and presentation quality as key to engagement regardless of setting.

**CONCLUSION:**

Residents had variable responses to the simulcast setting based upon their comfort with participation by group size, desire for social connection, and perception of teaching strategies. The key identified pitfalls to simulcast teaching were resident discomfort in small groups, heterogeneous learning experience, lack of engagement, and technology challenges. These pitfalls can be mitigated through strategic distribution of learners in groups, trained facilitators, and interactive teaching modalities. Given that simulcast and mixed (simulcast, virtual, and in-person) teaching settings are here to stay postpandemic, anticipating pitfalls and creating adaptable educational content that translates in multiple settings is crucial.

## Introduction

Medical educators around the globe adopted new technologies and teaching modalities to engage learners remotely during the COVID-19 pandemic.^1–3^ New challenges arose, such as the increased cognitive load of virtual education, and preexisting challenges were exacerbated, such as resident burnout.^4,5^ Graduate medical educators were called to maintain high educational excellence while supporting the health and well-being of their trainees in rapidly changing, overtaxed health systems.^6–8^ Although numerous online course delivery platforms existed prior to the pandemic, most were structured to fit traditional educational models and were not easily adaptable to graduate medical education (GME).^5^ In response to this educational challenge, to balance social interaction with exposure risk, we explored a simulcast model for GME. In this model, residents were divided into small groups that met physically in separate rooms and connected to a large-group session via videoconferencing.

We recognized the potential conceptual benefits of the simulcast model but found a paucity of data regarding learner response and optimal utilization of this model in GME. While interactive educational methods are often the most effective regardless of group size, small group learning is driven by discussion and highly dependent upon the communication skills and group dynamic of the facilitator and learners.^9,10^ Simulcast methods have been used in undergraduate and graduate educational programs but few studies in healthcare education report on learner response to this model.^11^ Existing studies report varied perceptions. In one study from 2010, radiology residents in a program that used simulcast didactics across 8 geographically separated practice sites had positive perceptions overall, with 71% reporting they would not have been able to participate without videoconferencing.^12^ There was, however, dissatisfaction with the visual quality and interactive discussions between centers.^12^ In another study from 2010 that assessed learning from videoconferencing in medical students who were colocated and dispersed, participants in a simulcast setting felt that interaction was effectively fostered even in groups without an in-person facilitator.^13,14^ We did not find any studies that directly compared virtual, in-person, and simulcast models, and technology has evolved since these examples. Thus, we sought to further define the perceived advantages and disadvantages of learning in simulcast settings in GME.

In this qualitative study, we explored feasibility and residents’ perceived advantages and disadvantages of learning in simulcast settings compared to virtual and in-person settings during ambulatory educational conferences during the COVID-19 pandemic. The results of this study can guide graduate medical educators as we continue to navigate mixed virtual, in-person, and simulcast settings postpandemic.

## Methods

### Overview

This qualitative study used inductive thematic coding to analyze the experiences of internal medicine (IM) residents who participated in an ambulatory curriculum in simulcast, virtual, and in-person settings.

### Setting

The IM residency program at the Penn State Health Milton S. Hershey Medical Center includes 60 categorical IM residents who rotate through a 6 + 2 block model. During their 2-week ambulatory blocks, 14 to 17 residents attend a weekly, mandatory 3-hour academic half-day during which faculty facilitators and resident educators cover a variety of ambulatory topics. During the 2020-2021 academic year (study period July 1, 2020 to June 30, 2021), we transitioned between simulcast, virtual, and in-person teaching models due to the COVID-19 pandemic. By the end of the academic year, all participants had experienced all three educational models: the year started with all learners having virtual teaching at the peak of the pandemic, transitioned to simulcast teaching mid-year, and then transitioned to larger group in-person teaching by the end of the year.

In the simulcast model, 3 groups of 4 to 6 residents met in-person in separate rooms and connected via videoconferencing (simulcast intervention groups). Group size of 4 to 6 residents was chosen based upon existing observations of group size for effective small teaching and available room space to accommodate social distancing.^10^ The small groups were assigned and designed to include learners from all levels of training. The lead facilitator was physically present in one small group room. We quickly found that learners in the rooms without the lead facilitator were less engaged compared to learners in the room with the lead facilitator. We therefore recruited an additional dedicated facilitator (chief or post-graduate year [PGY] 3 resident with an interest in medical education) for each room. These additional facilitators were emailed the teaching session materials and a description of their role prior to the session. Core faculty and chief residents met regularly to provide feedback and discuss encountered challenges.

Existing educational materials were already designed to have high interactivity (eg, case-based, gamification, worksheets, discussion) and were adapted to accommodate the changes in learning setting. For example, questions prompting discussion in a large group case could be divided and optimized for smaller group simulcast learning.

### Participants, eligibility criteria, and recruitment

Eligible participants were IM residents at Penn State who participated in the ambulatory half-day curriculum during the 2020-2021 academic year. Individuals who were not IM residents at Penn State in this curriculum were not eligible to participate.

Via e-mail, we invited all eligible residents to participate in focus groups during their ambulatory block in late Spring of the academic year (once all residents had experienced all 3 teaching settings). Nonresponders were sent one additional email after 1 week. Information on study purpose and confidentiality was emailed to volunteers prior to the focus group and reviewed at the start of each focus group. Verbal consent was obtained and recorded at the start of the focus group for each participant.

### Instrument development

The research team developed a semistructured interview guide prior to study initiation focused on residents’ perceived advantages and disadvantages of learning in various settings during the pandemic (Appendix 1). We developed this guide based on the experience of the study authors (RC, JC, and EH) as well as 2 pilot interviews with residents prior to the start of the study. SG reviewed the completed guide for clarity and content. The interview guide was not formally validated.

### Data collection

Eligible participants (*N* = 14 to 17 residents each block) were invited to participate in one 30-min audio-recorded virtual focus group during their ambulatory block. A total of 5 focus groups, each comprised of 2 to 9 residents who agreed to participate each block, were completed in April to June 2021 (interview groups). The focus groups were led by a faculty member with experience in focus group facilitation who was not involved in the teaching or assessment of the IM residents (SG).

### Data analysis

We followed the Framework Method for thematic analysis.^15^ Audio files of the focus groups were transcribed verbatim with all identifiers removed. We then read the transcripts line-by-line to identify relevant codes representing voiced concepts. Three investigators (RC, JC, and EH) independently coded three transcripts and developed the initial list of coding categories by consensus. Additional transcripts were independently coded by 4 investigators (RC, JC, EH, and SG) with modifications to the codebook until an interrater reliability reached a kappa score of 0.7 for 3 interviews. All interviews were subsequently analyzed using the finalized codebook (Appendix 2) using NVivo (released March 2020). We then collaboratively developed representative themes using inductive thematic coding. Demographics were reported using descriptive statistics. While we collected focus group data on residents’ experiences with teaching in the various models, we did not find sufficient codes in this area to draw thematic conclusions (Appendix 2). Saturation was otherwise reached during thematic analysis.

This study was approved by the Institutional Review Board at the Pennsylvania State University (Study ID 17279).

## Results

We found that residents had variable perceptions of simulcast, in-person, and virtual teaching settings based upon their preferences for group size, physical presence, social connection, and flexibility. While our residents generally preferred in-person teaching, they reflected that learning could be engaging in any setting with interactive teaching techniques.

### Feasibility

We fortunately had accessible facilities and equipment to teach in virtual and simulcast settings at no additional cost. Support for room scheduling and resident notification about teaching location by administrative staff and chief residents was crucial. We found that the training needed for facilitators was not overly time intensive, however, our teachers were mainly a core group of faculty and chief residents throughout the year.

### Participant characteristics

Forty-eight percent (*n* = 29/60) of invited residents participated in focus groups. Of the participating residents, 45% (*n* = 13) were female and 55% (*n* = 16) were male. All 3 training years were represented (38% [*n* = 11] PGY1, 41% [*n* = 12] PGY2, 21% [*n* = 6] PGY3).

### Advantages and disadvantages of learning by setting

The perceived advantages and disadvantages of learning in the simulcast, in-person, and virtual settings are summarized in Table 1.

**Table 1.** Advantages and disadvantages of learning by setting.

Setting	Advantages	Disadvantages	Quote
Simulcast	Ability to intentionally create groups with mixed learner levels Increased engagement due to accountability to peers Facilitation of a safe and collaborative learning climate Increased engagement of quieter learners Learning through informal peer discussions Increased interactivity	More challenging to interact with facilitator if in outside room Challenge of both navigating technology and finding physical room Variable experience based upon people in small groups and facilitator	It (simulcast) was kind of a lower pressure. Everyone's just kind of collaborating and brainstorming together, and I think that that created a safe learning environment. If everyone kind of is of the opinion that they don't really want to do much and participate, then that kind of trickles down and then it's less interactive.
In-person	Ease of involvement in group discussion Learning through Informal conversations Relationship building Improved concentration Enhanced communication through nonverbal cues Flexibility to adapt time to needs of group	Getting to geographic location Noise/distractions with large group Feeling uncomfortable speaking in large group	You have these little side conversations that help your learning with your peers and also with the instructor or the person who's giving the presentation. I think when I'm in a large group, it's easier for me to kind of zone out even if it's like a discussion, because there are just so many people.
Virtual	Conducive to life/work integration Increased engagement of quieter learners Convenient Ability to electronically capture and save high-yield presentations Ability to use break-out rooms	Distractions in home and virtual environment Difficult to remain focused without in-person connection Lack of social cues to guide responses More difficult to interact with peers/facilitator	I have seen a lot of people who don't like to speak much, including myself, you can express your thoughts easily through Zoom. I think if you're just kind of alone at home, you're not really engaged because you can turn off the camera or you can kind of half participate.

**Figure 1.**
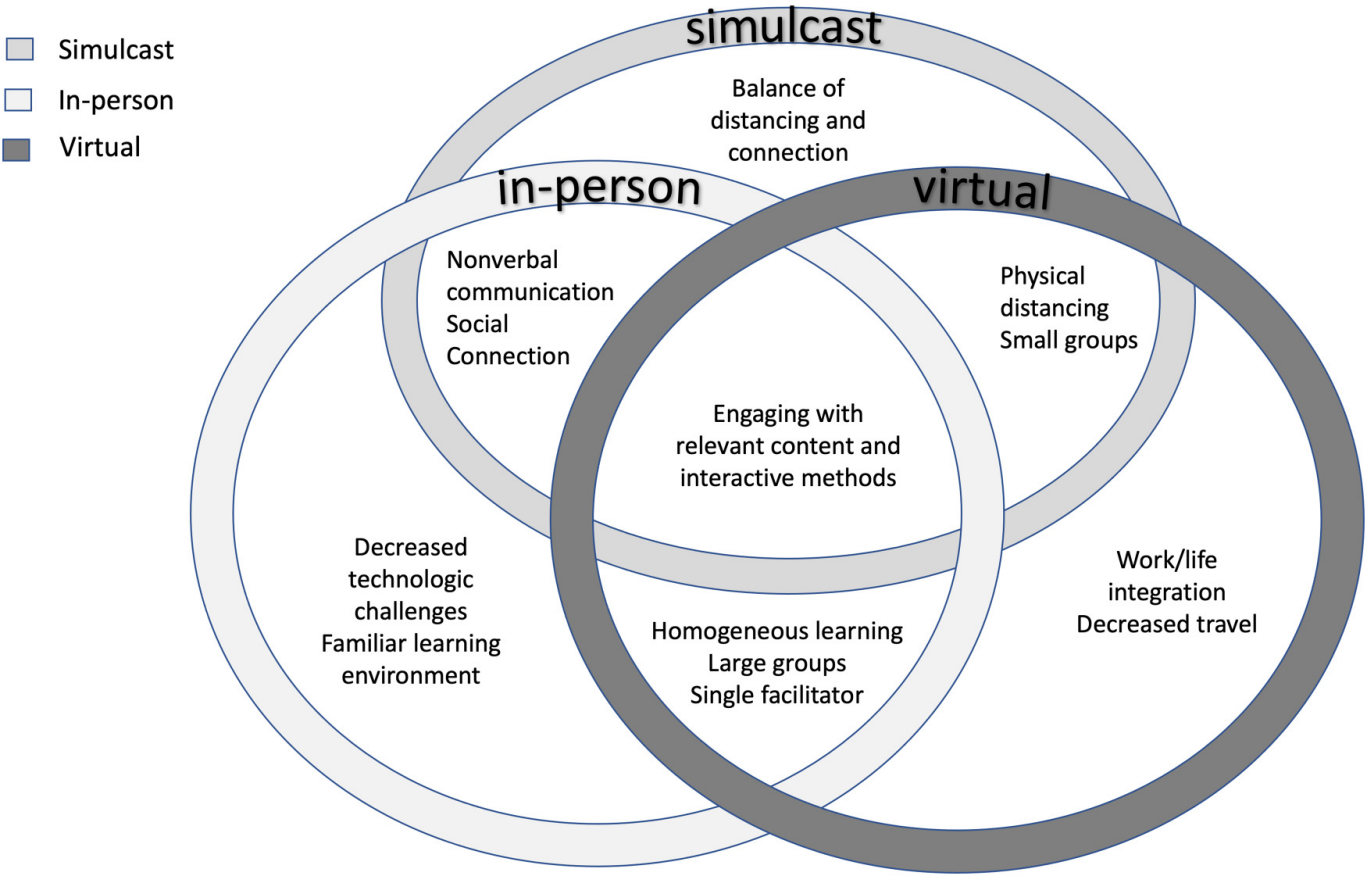
Intersectionality of advantages of learning in in-person, virtual, and simulcast settings.

For the simulcast and in-person settings, residents valued social connection and the ability to recognize nonverbal cues from other participants. The virtual meetings were convenient and allowed residents to multitask while at home, but this flexibility was also viewed as a potential distractor. The simulcast setting posed novel challenges including technical difficulties with videoconferencing across multiple rooms and variable intergroup experiences depending on the composition of the learner group and the facilitator. The intersectionality of perceived advantages between the three settings are further illustrated in Figure 1.

The level of engagement in each setting is varied by individual resident. In the simulcast setting, participants expressed a sense of camaraderie and accountability (or personal obligation) to participate in their small group, particularly for those who felt less comfortable in larger group settings. Residents expressed that they received a heterogeneous learning experience based upon the other learners and educators in their room, particularly if they were not in the room with the main session facilitator. Some residents reported that it was easiest to participate in-person because they could hear and concentrate better, while others felt distracted and hesitant to speak in the large group. In the virtual setting, quieter learners appreciated the ability to participate via chat, while others felt lonely and less engaged.

### Preferred setting for learning

In the absence of restrictions imposed by the pandemic, participants predominantly preferred the in-person setting (11 comments) for learning. The simulcast model was acknowledged by some participants (5 comments) as the optimal option during the restrictions of the pandemic, due to the balance of social distancing with in-person interaction. Participants proposed a fourth model to balance engagement and flexibility, in which session facilitators would present in-person, but residents would have the option to participate in-person or virtually.

Overall, residents expressed that the setting was less important than the preparation, enthusiasm, and skill of the educator. Technology and engagement hurdles could be overcome:

*it (the setting) doesn't make a huge difference as long as they utilize the technology well, and as long as they're well prepared with a good presentation…*

A “good” presentation was well-focused with relevant content. They valued the use of interactive teaching methods regardless of the setting, and felt that teaching strategies could be adapted to suit the needs and challenges of each model. For example, effective use of virtual break-out rooms could be as engaging as the simulcast in-person rooms. Additional suggested techniques included annotation, surveys, and gamification. Utilization of strategies to enhance interactivity was reported to be particularly important when learners were not physically present in the same room as the main facilitator in the simulcast setting. For example, repeating audience questions and the correct response was suggested to standardize the experience across all learner groups.

## Discussion

In response to the COVID-19 pandemic, our IM residency program piloted simulcast educational sessions to balance participant engagement and social interaction with infection exposure risk. We found that residents had variable perceptions of the simulcast setting compared to in-person and virtual settings based upon their prioritization of social connection and flexibility. Heterogeneous experiences by simulcast groups may be overcome with interactive techniques by trained facilitators.

The advantages and disadvantages of the simulcast models identified by our residents are aligned with the limited prior studies of learners in these settings. In nonmedical education, the simulcast model has been shown to create a more flexible, engaging learning environment compared to virtual or in-person instruction.^9^ Our learners echoed that the simulcast model, however, has both pedagogical and technological challenges and relies on skilled educators to manage the added complexity.^9^ Despite these challenges, our residents valued the interactive small group activities that increased engagement in both the virtual and simulcast settings.

Although residents in our study recognized the advantages of the simulcast model during the pandemic, they still generally preferred a larger group in-person setting when possible. Multiple factors may have contributed to this preference. In-person sessions are the traditional and most familiar mode of delivery for resident didactics. Residents were faced with social isolation both inside and outside of the workplace during the pandemic, and placed high value on social interaction. While the simulcast model allowed for some social connection, learning in this setting was perceived as heterogeneous across groups. While we attempted to minimize variation across rooms by using consistent, trained facilitators and summarizing key small group learning points, it was difficult to ensure a uniform learning experience. Additionally, there were challenges in navigating unexpected technological troubles in separate rooms that may have counteracted the identified advantages.

### Lessons learned

Simulcast teaching modalities, particularly mixed with virtual and in-person teaching settings, will continue to be frequently utilized in the postpandemic setting. For example, our institution wide “virtual” resident conferences frequently include learners physically together in a main room with the facilitator, individuals in a virtual platform, and groups of learners in rooms together signed in virtually. Pitfalls and recommendations for best practices in simulcast teaching based upon our experiences and the resident perceptions outlined in this study are summarized in Table 2.

**Table 2.** Key strategies for simulcast teaching.

Pitfall	Best practice to overcome pitfall
Resident discomfort in small groups	Set expectations for small group participation Encourage a positive learning climate in small groups (eg, ice-breaker for all groups and time to discuss) Alternate the “spokesperson” who responds following small group activities Strategically distribute learners in groups with different engagement styles and learning levels
Heterogeneous learning experience by group	Trained facilitators (faculty or trainee) in each small group room Repeat all questions and answers (particularly those made in the group with the main facilitator) Mix learner groups between sessions and/or have the lead facilitator alternate between rooms
Lack of engagement	Design interactive activities optimized for small groups (eg, case-based learning, team-based learning [TBL]) Mix small group and individual level activities (eg, audience response) Have each small group alternate reporting responses to the main facilitator
Technology challenges	Provide enough time before sessions start to troubleshoot issues Experiment with technology before sessions in each room which will be utilized Have a dedicated participant in each room for set-up (computer/virtual platform sign-in, audiovisual display)

We have found that teaching in simulcast and “mixed” settings (main group is in-person together with a few virtual, individual learners) can be challenging to navigate, but has advantages over purely virtual or in-person settings. For mixed virtual and in-person settings, the use of engagement techniques that work equally well for individuals and groups across both settings are crucial (eg, case-based learning and audience response), as are the techniques outlined in Table 2 which ensure that all learners have as equitable an experience as possible. Our curriculum was enhanced by the creation of a core set of materials that could be adapted to multiple teaching settings, while preserving their objectives and interactivity. This aligns with our residents’ overall perception that the engagement of the session and the facilitator were more important than the setting. Facilitator skills and experience in small group teaching, however, can vary. For our more junior facilitators, their role was mainly to enhance accountability of the group. With this in mind, we found an advantage of the simulcast setting compared to unconnected small groups was that one experienced educator was always present to navigate teaching challenges and answer questions.

### Limitations

Our data is limited to resident experiences from a single academic institution during the first year of the COVID-19 pandemic. While participants experienced all 3 models, they had more familiarity and longer-term experience with the traditional in-person sessions. We acknowledge that the residents’ experiences with virtual and simulcast learning occurred early in the pandemic when we were still establishing virtual culture and adapting to virtual teaching techniques. The focus groups were offered at the end of 2021 and asked for comparisons which may not fully reflect the residents’ perceptions at the time of initial implementation, or the current views of residents postpandemic. While we interviewed a balanced sample of residents by sex and year in residency, we do not know if perceptions differed by these groups as participant data was deidentified. We acknowledge that learner preference is only one component of evaluating educational sessions, and that we did not measure changes in learner skills or knowledge, or clinical outcomes. Simulcast small groups were facilitated by educators with varied training and experience, which may have impacted engagement. Because this was a convenience sample of participants, we did not calculate sample size. Our interview guide was not previously validated.

### Future directions

Additional studies with deliberate comparison groups could further delineate the educational effectiveness of simulcast compared to virtual and in-person settings. This could be helpful to programs designing effective education for geographically disparate learner groups, or for interventions across institutions that allow for shared resources and expertise. Future studies could explore noneducational effects of these settings, such as burn-out, social connection, and professional identity, and the alignment between educator and learner perceptions of the advantages, challenges, and effectiveness of these settings.

## Conclusion

While the simulcast model was viewed favorably in the unique pandemic circumstances to balance education, social interaction, and safety, these benefits were mitigated by technology challenges and a heterogeneous learning experience. As simulcast and mixed teaching settings are here to stay, anticipating pitfalls and creating interactive, adaptable educational content that translates in all settings is crucial.

## Supplemental Material

sj-docx-1-mde-10.1177_23821205241281350 - Supplemental material for Resident Perceptions of Simulcast Teaching: A Qualitative Study Supplemental material, sj-docx-1-mde-10.1177_23821205241281350 for Resident Perceptions of Simulcast Teaching: A Qualitative Study by Rachel S. Casas, Jennifer L. Cooper, Susan A. Glod and Eliana V. Hempel in Journal of Medical Education and Curricular Development

sj-docx-2-mde-10.1177_23821205241281350 - Supplemental material for Resident Perceptions of Simulcast Teaching: A Qualitative Study Supplemental material, sj-docx-2-mde-10.1177_23821205241281350 for Resident Perceptions of Simulcast Teaching: A Qualitative Study by Rachel S. Casas, Jennifer L. Cooper, Susan A. Glod and Eliana V. Hempel in Journal of Medical Education and Curricular Development
