# Supplementary material for: Resident Perceptions of Simulcast Teaching: A Qualitative Study
Source: J Med Educ Curric Dev. 2024 Sep 19;11:23821205241281350. doi: 10.1177/23821205241281350 (PMC11418307; doi:10.1177/23821205241281350)
Supplement: sj-docx-1-mde-10.1177_23821205241281350 - Supplemental material for Resident Perceptions of Simulcast Teaching: A Qualitative Study [file sj-docx-1-mde-10.1177_23821205241281350.docx]

Appendix 1: Semi-structured Interview Guide

1. How has the pandemic impacted your learning during ambulatory academic half day?
2. Can you tell me about what it has been like learning in simultaneous virtual and in-person sessions compared to virtual or in-person sessions?
   1. What advantages are to learning in these settings?
   2. What are challenges to learning in these settings?
3. How does your engagement as a learner differ in simultaneous virtual and in-person sessions compared to virtual or in-person sessions? Have you noted any differences in the engagement of other participants in these different settings?
4. Can you tell me about what it has been like teaching in simultaneous virtual and in-person sessions compared to virtual or in-person sessions?
   1. What are advantages to teaching in these settings?
   2. What are challenges to teaching in these settings?
5. If you could choose, would you prefer to learn in in-person, virtual, or simultaneous virtual/in-person ambulatory academic half day sessions?
6. If you could choose, would you prefer to teach in in-person, virtual, or simultaneous virtual/in-person ambulatory academic half day sessions?

Appendix 2: Qualitative Codes and Descriptions

| **Code** | **Description** | **Number of Files** | **Number of Coding References** |
| --- | --- | --- | --- |
| Impact of pandemic | Participant identified impact of the pandemic on teaching or learning | 2 | 5 |
| **Advantages of Learning by Setting** | | | |
| Advantages learning virtual setting | Participant identified advantages of learning in a virtual setting, including rationale for why this setting is advantageous | 4 | 15 |
| Advantages learning in-person setting | Participant identified advantages of learning in an in-person setting, including rationale for why this setting is advantageous | 4 | 9 |
| Advantages learning simulcast setting | Participant identified advantages of learning in a simulcast setting, including rationale for why this setting is advantageous | 5 | 23 |
| **Disadvantages of learning by setting** | | | |
| Disadvantages learning virtual setting | Participant identified disadvantages of learning in a virtual setting, including rationale for why this setting is disadvantageous | 5 | 19 |
| Disadvantages learning in-person setting | Participant identified disadvantages of learning in an in-person setting, including rationale for why this setting is disadvantageous | 3 | 4 |
| Disadvantages learning simulcast setting | Participant identified disadvantages of learning in a simulcast setting, including rationale for why this setting is disadvantageous | 4 | 10 |
| **Advantages of teaching by setting** | | | |
| Advantages teaching virtual setting | Participant identified advantages of teaching in a virtual setting, including rationale for why this setting is advantageous | 1 | 2 |
| Advantages teaching in-person setting | Participant identified advantages of teaching in an in-person setting, including rationale for why this setting is advantageous | 2 | 2 |
| Advantages teaching simulcast setting | Participant identified advantages of teaching in a simulcast setting, including rationale for why this setting is advantageous | 0 | 0 |
| **Challenges of teaching by setting** | | | |
| Challenges teaching virtual setting | Participant identified challenges of teaching in a virtual setting, including rationale for why this setting is challenging | 4 | 9 |
| Challenges teaching in-person setting | Participant identified challenges of teaching in an in-person setting, including rationale for why this setting is challenging | 1 | 1 |
| Challenges teaching simulcast setting | Participant identified challenges of teaching in a simulcast setting, including rationale for why this setting is challenging | 4 | 7 |
| **Comparisons across settings and learner preferences** | | | |
| Comparisons learning across settings | Comparisons of advantages, disadvantages, teaching methods, and/or engagement across settings (virtual, in-person, or simulcast) from the perspective of a learner | 5 | 26 |
| Comparisons teaching across settings | Comparisons of advantages, disadvantages, teaching methods, and/or engagement across settings (virtual, in-person, or simulcast) from the perspective of a teacher | 4 | 8 |
| Learner behavior while learning | Participant identified behaviors while learning | 3 | 12 |
| Preferred setting as learner | Participants’ preferred setting for learning (virtual, in-person, or simulcast) | 4 | 19 |
| Preferred setting as teacher | Participants’ preferred setting for learning (virtual, in-person, or simulcast) | 2 | 2 |
| Recommendations for engaging learners | Overall recommendations for engaging learners in any setting | 5 | 36 |
